# Synthesis and Applications of Carboxymethyl Cellulose Hydrogels

**DOI:** 10.3390/gels8090529

**Published:** 2022-08-24

**Authors:** Wenliang Zhang, Yining Liu, Yang Xuan, Shubiao Zhang

**Affiliations:** Key Laboratory of Biotechnology and Resource Utilization of Ministry of Education, Dalian Minzu University, Dalian 116600, China

**Keywords:** carboxymethyl cellulose, hydrogel, functional, drug carrier

## Abstract

Hydrogels are basic materials widely used in various fields, especially in biological engineering and medical imaging. Hydrogels consist of a hydrophilic three-dimensional polymer network that rapidly expands in water and can hold a large volume of water in its swelling state without dissolving. These characteristics have rendered hydrogels the material of choice in drug delivery applications. In particular, carboxymethyl cellulose (CMC) hydrogels have attracted considerable research attention for the development of safe drug delivery carriers because of their non-toxicity, good biodegradability, good biocompatibility and low immunogenicity. Aiming to inspire future research in this field, this review focuses on the current preparation methods and applications of CMC gels and highlights future lines of research for the further development of diverse applications.

## 1. Introduction

Hydrogels, also called colloid gels, are three-dimensional cross-linked materials comprising water-soluble polymers, with water as the dispersing medium [1,2]. Initial research on hydrogels focused on their basic characteristics, including swelling equilibrium, swelling dynamics, solute diffusion, volumetric phase transition and sliding friction [3,4]. This led to the discovery of their excellent features of high water content, high porosity and soft consistency, which have resulted in hydrogels being widely used as the closest mimic of natural tissue in synthetic biomaterials [5,6]. In addition, hydrogels can be formulated into various physical forms, including plates, particles, nanoparticles, coatings and films. Therefore, hydrogels have a wide range of applications in experimental and clinical medicine, including tissue engineering [7,8], regenerative medicine [9], diagnostics [10], cell immobilisation [11], biomolecular [12] or cell separation and barrier materials for regulating biological adhesion [13,14].

Carboxymethyl cellulose (CMC) is an anionic polymer compound with a molecular weight of thousands to millions Da, having a white fibrous or granular powder with odourless, tasteless and hygroscopic characteristics [15]. Interestingly, CMC tends to disperse in water to form a transparent colloidal solution, i.e., CMC gel. Owing to its low toxicity and immunogenicity, intelligent responsiveness [16] and good biodegradability and biocompatibility [17], CMC gels have attracted extensive research attention [18], becoming one of the most promising materials for application as drug carriers in clinical fields.

Hydrogels can be prepared via chemical cross-linking and physical cross-linking of polymers [19]. In chemical cross-linking, hydrogels are formed by adding cross-linking agents into a polymer aqueous solution [20,21], whereas physical cross-linking occurs through electrostatic interaction, ionic interaction, hydrogen bonding, chain winding and other physical forces. Due to the increase in hydrogel research, researchers have shifted their focus from ordinary hydrogels to smart responsive hydrogels. At this stage, various smart hydrogels that can respond to the changes in environmental conditions, including temperature, electric field, pH or other conditions, have been developed. Consequently, the synthesis of CMC gels has attracted increasing attention. This review mainly summarized the preparation methods of CMC gels through three categories: cross-linking by metal ions, radiation cross-linking and cross-linking with other natural polymers. Some other important preparation methods, such as those obtained by free radical polymerization, are not described in detail. Furthermore, the applications, future research directions and prospects of CMC gels are discussed. We hope this review will inspire more research on the preparation and applications of hydrogels.

## 2. Preparation of Carboxymethyl Cellulose Hydrogels

### 2.1. Cross-Linking by Metal Ions

The reaction of CMC with metal ions is one of the most common chemical cross-linking strategies for synthesising CMC gels [22,23,24]. In this method, as the cross-linking occurs via the formation of chemical bonds, the generation of the CMC gel is irreversible.

**Figure 1:**
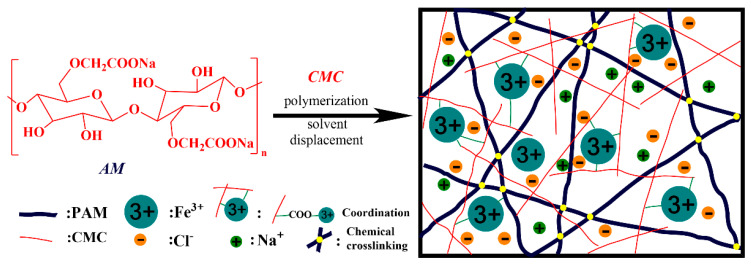
Preparation process and internal structure of PAM/CMC organohydrogel [25]. Copyright 2019 Elsevier Ltd.

Using this approach, gel-based sensors have been synthesised and extensively investigated due to their flexible and extensible properties. Duan et al. prepared an excellent anti-freezing and mechanical (fracture energy of 5238 kJ/m^3^) CMC-gel sensor using Fe^3+^-cross-linked CMC as the first network and covalently cross-linked polyacrylamide (PAM) as the second network (Figure 1) [25]. The porous network structure favoured the transportation of Fe^3+^ through the water channels upon the ionisation of FeCl_3_ in the binary solvent, endowing the gel sensor with a quick response (0.98 s) and high sensitivity (gauge factor = 1.4, 0–30% strain) to realise subtle monitoring in the human body. The resulting CMC gel with quick response and high sensitivity exhibited great potential for application in next-generation biological-sensing materials and devices.

In addition to Fe^3+^, Al^3+^ is a common and important cross-linking agent [26]. Using Al^3+^ as the cross-linking agent, Liu et al. successfully synthesised a CMC gel [27]. Upon dissolving CMC powder in water, the free segments of CMC entangled with each other. The viscosity increased and the flow index decreased with increasing CMC content. The thin surface of the CMC gel was dissolved when the gel was added into Al_2_(SO_4_)_3_ solutions. The carboxymethyl groups of CMC were ionised into negative ions, which were then combined with Al^3+^ via chemical bonds to form a thin reticular film on the CMC-gel surface. The cross-linking films could prevent the dissolution of CMC in water and lock the free water molecules inside to form a hydrogel. Infrared spectroscopy and thermogravimetric analyses showed that the light transmittance of the CMC gel improved significantly upon increasing the amount of CMC substitution. Moreover, the swelling rate of the CMC gel increased with increasing temperature and the substitution amount of carboxymethyl groups.

Metal-ion doping can not only promote the formation of hydrogels, but also change their properties [28]. Shao et al. obtained bulk Al-doped CMC gels via a facile slow-gel method and freeze-drying technology [29]. The reaction rate between carboxyl groups and Al^3+^ was controlled by controlling the release of Al^3+^. Because of the steady reaction rate, Al^3+^ was well-distributed in the gels via bidentate bridging coordination between Al^3+^ and CMC. By adjusting the content of Al^3+^ and the degree of substitution of CMC, a crossing network and tightly packed sheet structure were obtained. Thus, the resulting CMC gels showed excellent load-bearing characteristics and outstanding flame retardation. This review offers a worthy reference for the synthesis process and application of CMC gels.

Some studies have shown that the pH of the solution varies with the type of metal salts. In addition, the properties of the CMC gels may vary with the degree of substitution in a CMC solution and even with the mixing sequence and mode of the CMC solution and salt. Therefore, the preparation of CMC gels by metal-ion cross-linking is challenging and promising.

### 2.2. Radiation Cross-Linking

The term radiation cross-linking refers to the formation of chemical bonds between the main chains of linear molecules upon irradiation [30,31]. This method avoids the addition of a cross-linking agent, ensuring no chemical doping, which not only reduces the complexity of the reaction and the production of by-products but also ensures the non-toxicity of the resulting hydrogel.

Wach et al. investigated the radiation cross-linking of CMC with a degree of substitution (DS) of 0.7–2.2 by irradiating CMC in the solid state and aqueous solutions at various irradiation doses with a γ ^60^Co source [32,33]. The results showed that the concentration and the DS of the solution had a significant effect on the cross-linking of CMC. By irradiating 20% of CMC content in a 5% aqueous solution, CMC with a DS of 2.2 induced higher cross-linking than that with a DS of 1.32. In addition to high DS, a high concentration in aqueous solution favoured the CMC cross-linking. This method is simple to operate, and the cross-linking degree can be controlled by changing the radiation conditions and monomer concentration. Moreover, as no initiator is required, a purer and non-toxic product can be obtained in one step.

Ultraviolet (UV) light can also be used as the radiation source to prepare CMC gels [34,35]. Dadoo et al. synthesised norbornene-modified CMC (NorCMC) successfully by functionalising CMC with norbornene groups via 1-ethyl-(3-dimethylaminopropyl) carbodiimide hydrochloridecoupling reaction. Then, through photoinduced thiol–norbornene reaction, NorCMC hydrogels with dithiol molecules were fabricated with highly tuneable compression moduli ranging from 2 to 103 kPa. The hydrogel modulus could be controlled by changing the UV irradiation time or other conditions. A series of experimental results demonstrated that NorCMC is a type of biomaterial with a variety of biomedical applications [18].

However, the radiation cross-linking method is not widely used because it has several drawbacks. For instance, the forces between the molecules of a physically cross-linked gel are reversible and could be broken by varying the pressure or other physical conditions. The yield of irradiated polymers in an aqueous solution is affected by diverse factors, including the polymer concentration, temperature, pH, dose rate, radiation dose and the presence of various metal ions and oxygen. Moreover, radiation may destroy some of the physiological properties of the materials.

### 2.3. Cross-Linking with Natural Polymers

In the metal-ion cross-linking method, the properties of the obtained gels depend on several factors, such as the type of metal salts, solution pH [36], degree of CMC substitution and mixing sequence and mode of CMC solution and salt, which has significant advantages and disadvantages. Moreover, as mentioned above, radiation polymerisation may destroy some physiological characteristics of the hydrogel and suffer from several uncontrollable factors. In contrast, hydrogels prepared via cross-linking with a natural polymer exhibit good biodegradability and mild and controllable reaction conditions, which render this approach as the most ideal and promising synthesis method for CMC gels [37,38]. At present, the use of environmentally friendly natural polymers is one of the main directions in the pursuit of a modern society. The CMC gels obtained by co-polymerising two types of natural polymers and using biodegradable additives have good biodegradability and are receiving unprecedented attention as a new type of medicinal material [39].

CMC gels are generally super-absorbent and highly biosafe. However, their applications are limited due to their low mechanical strength. To overcome these shortcomings, Jung et al. used a biocompatible natural polysaccharide, bacterial succinoglycan (SG), to synthesise new inter-penetrating polymer network (IPN) hydrogels in a non-bead form via a double cross-linking strategy [40]. These novel SG/CMC-based IPN hydrogels exhibited high mechanical strength while retaining the super-absorbent characteristic of CMC hydrogels. Moreover, SG/CMC gels exhibited pH-response drug release for drug and exhibited no cytotoxicity to HEK-293 cells, suggesting their potential application as future biomedical biomaterials for drug delivery.

**Figure 2:**
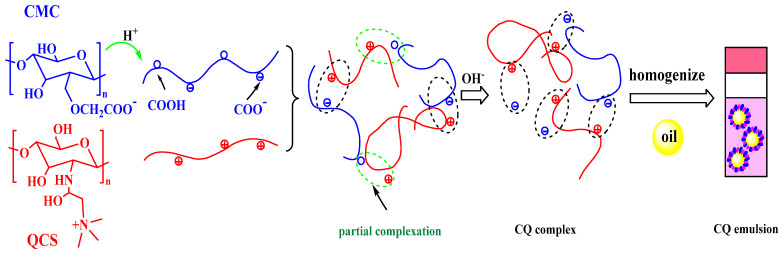
Scheme for the formation of the CQ complex [41]. Copyright 2021 Elsevier Ltd.

Zhu et al. synthesised a CMC and quaternary chitosan (QCS) complex to produce Pickering emulsions [41]. After partial protonation, CMC was mixed with QCS and neutralised at pH 7 to produce CMC/QCS (CQ) particles (Figure 2). The prepared CD not only had good long-term stability but also effectively reduced the degradation rate of curcumin. This research provided a stable and simple novel method for synthesising the Pickering emulsion delivery system. Furthermore, it provided a new application prospect for CMC gels that have potential application value in the fields of drug delivery and food production.

Wang et al. synthesised cyclodextrin-grafted cellulosic hydrogel beads (CD1@HEC-CMC-gel) through electrostatic and host–guest interactions [42]. The electropositive binding site used quaternary ammonium groups modified-Cyclodextrin (CD1), whereas electronegative binding site used CMC gels. By controlling the cross-linking between hydroxyethyl cellulose (HEC) and CMC, a double-network structure was synthesised in an epichlorohydrin environment. CD1 was grafted onto the electronegative double-network structure via electrostatic interactions, which allowed reaching a grafted CD1 content of 93.10±0.74%. After dialysis in water for 48 h, the grafted CD1 exhibited enhanced stability. Meanwhile, the host–guest interaction promoted the assembly of hydrophobic ibuprofen in the hydrophobic cavity of CD1 with an assembly ratio of 82.56%, as determined by UV absorption, which endows the IBU/CD1@HEC-CMC gel with pharmaceutical potential.

## 3. Applications

### 3.1. Anti-Tumor Application

**Figure 3:**
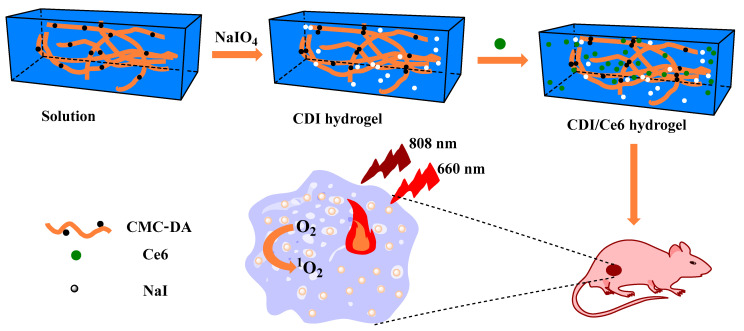
Schematic illustration of the preparation of CDI/Ce6 hydrogel for photothermal and photodynamic anti-tumor recurrence [48]. Copyright 2021 Elsevier Ltd.

Drug delivery vector is one of the most common applications of CMC gels owing to their good drug loading and drug release ability [43,44,45,46,47]. Zhang et al. designed a type of CMC gel, CMC-polydopamine-NaI (CDI) hydrogel, with the anti-tumor abilities of photothermal therapy (PTT) and photodynamic therapy (PDT) after loading polydopamine (PDA) and Chlorin e6 (Ce6) [48]. The addition of sodium periodate (NaIO_4_) had two functions. One was promoting the formation of CMC gels and the other was oxidising dopamine to PDA (Figure 3). In addition, NaIO_4_ was reduced to sodium iodide to realise computerised tomography imaging for monitoring the process of hydrogel degradation and tumor therapy. Furthermore, PDA could increase the temperature and Ce6 could produce cytotoxic reactive oxygen species under near-infrared light irradiation. Thus, combined treatment of tumors could be achieved. Hence, the side effects of the photosensitiser in healthy tissues can be avoided using the gel system, which laid the foundation for the rational design of biodegradable hydrogels for multi-functional applications.

Ph-sensitive spray-dried microspheres prepared from a polyacrylamide–sodium carboxymethyl cellulose (PAAMG–CMC–Na) co-polymer can be used for colon-targeted delivery. The microspheres prepared by Vijaykumar et al. using natural CMC–Na as a carrier could not prevent the drug release in the stomach and small intestine [49]. However, upon grafting the microspheres onto the PAAmg polymer to improve the CMC–Na function and converting the amide functional group of PAAmg to a carboxyl group, the CMC–Na co-polymer became pH-sensitive. The microspheres prepared by cross-linking the pH-sensitive PAAmg–CMC–Na co-polymer with glutaraldehyde could block the drug release in the stomach and small intestine, increase the drug release amount in the cecum, achieve colon-targeted delivery and accelerate the cure time.

Sangsuriyonk et al. showed that CMC was also a potential bio-based matrix for electrically controlled non-ionic drug delivery [50]. CMC-based drug-matrix hydrogels were prepared through solution casting with different concentrations of citric acid as the crosslinker.

A bismuth-embedded nanohydrogel (Bi@CMC) was synthesised under UV irradiation by Zhao et al. A series of characterisations revealed that Bi@CMC had good characteristics of imaging and PPT [51]. Besides, Bi@CMC could integrate medical imaging and PPT/drug dual-mode therapy after adsorption of doxorubicin. Moreover, the nanohydrogel was disintegrated after irradiation and metabolised more easily, thereby reducing side effects and toxicity. This study proposed a new dual-mode bioimaging and chemo-photothermal combined therapy nanoprobe and proved the potential of CMC gels for use in medical imaging and as a drug carrier.

### 3.2. Anti-Microbial Application

In recent years, CMC has been widely used in different fields because of its excellent properties, including good biocompatibility, biodegradability, non-toxicity and low cost. In addition to the early diagnosis and treatment of tumors, CMC gels also have applications in bacteriostatic fields [52,53].

Du et al. oxidised adjacent hydroxyl groups on CMC to aldehyde groups, which were then subjected to a Schiff-base reaction with the amine groups on carboxymethyl chitosan to produce pH-sensitive hydrogels and their microgels composites [54]. The gelation time, weight loss ratio, swelling ratio, morphologies, mechanical properties, pH-sensitive drug release profiles and antibacterial activities of the hydrogel complexes were examined using silver sulfadiazine as an anti-microbial model drug, achieving satisfactory antibacterial activity that could be used in wound dressings in the future.

Jeong et al. designed and synthesised a dual-component system consisting of both carboxymethyl β-cyclodextrin (cmβCD) and CMC [55]. Tetracycline was used as a drug model to study drug-loading efficiency and controlled release characteristics. A series of experiments proved that the cmβCD/CMC hydrogels were less toxic, biocompatible and can be effectively used in drug delivery systems.

### 3.3. Other Applications

In addition to their application in medical fields as anti-tumor and bacteriostatic agents, hydrogels have applications in agriculture [56,57,58], wound healing [59] and other fields [60,61,62,63].

For instance, Pulat et al. synthesised CMC and carrageenan hydrogels for growing wheatgrass plants [64]. The treatment of soil with the CMC gels not only enhanced the water retention capacity of the soil but also promoted the growth of wheat by loading zinc micronutrient. These results suggest that hydrogels can be used as a controlled fertiliser system in agricultural fields.

In addition to promoting plant growth, CMC gels can be used for pesticide release. Sarkar and Singh synthesised a boric acid-cross-linked CMC gel to develop pH-responsive release formulations of boron and thiamethoxam [65]. Entrapment of thiamethoxam was conducted via ex situ loading. These types of CMC gels may be useful in the selective release of nutrients and pesticides in different soils.

## 4. Outlook

At present, cancer still presents a high incidence and mortality. Therefore, the development of efficient cancer treatments is a pressing concern. Despite being a widely used and accepted clinical treatment, chemotherapy poses serious disadvantages, such as side effects and the development of drug resistance of tumor cells. The use of drug carriers has emerged as an effective approach to solve these problems. Among the developed nanocarriers, CMC gels have attracted the attention of researchers due to their low cost, high drug-loading efficiency and low toxicity. In addition, CMC gels can maintain drug concentration at a pre-set level for a long time through a slow release, thereby reducing drug toxicity and side effects. Using CMC gels loaded with drugs, effective inhibition of cervical cancer, liver cancer, human glioma and other malignant tumors has been achieved. Chemotherapy often requires complementation with other treatments such as photothermal therapy, photodynamic therapy or immunotherapy to realise the synergistic treatment of tumors. As CMC gels can load a photosensitiser and chemotherapy drugs simultaneously, it seems plausible that CMC gels will play an important role in the treatment of tumors. In addition, further research will certainly lead to the application of CMC gels in various applications such as wound dressings [66] and agricultural activities [67]. Further optimisation of the synthetic methods will indicate the new properties of CMC gels, which will broaden their application scope.

In the future, the application prospect and scope of CMC gels can be considered from the following two aspects:

1. Intelligent and responsive CMC gels should be developed further. Using the tumor microenvironment or external stimulation [68], the time and space control of chemotherapy drugs could be achieved easily, which can not only reduce the side effects but also achieve real-time tumor monitoring.
2. CMC gels could be modified to adapt to gene therapy. The most popular gene editing and therapy vectors are liposomes and lipid nanoparticles. However, these two vectors still have disadvantages. If CMC gels can be better used for gene delivery via modification, CMC gels will have broader applications.

## Data Availability

Not applicable.
